# Ultra-highly sensitive dual gases detection based on photoacoustic spectroscopy by exploiting a long-wave, high-power, wide-tunable, single-longitudinal-mode solid-state laser

**DOI:** 10.1038/s41377-024-01459-5

**Published:** 2024-05-01

**Authors:** Shunda Qiao, Ying He, Haiyue Sun, Pietro Patimisco, Angelo Sampaolo, Vincenzo Spagnolo, Yufei Ma

**Affiliations:** 1 National Key Laboratory of Laser Spatial Information, Harbin Institute of Technology, Harbin, China; 2 PolySense Lab, Dipartimento Interateneo di Fisica, University and Politecnico of Bari, Via Amendola, Bari, Italy

**Keywords:** Infrared spectroscopy, Optical sensors

## Abstract

Photoacoustic spectroscopy (PAS) as a highly sensitive and selective trace gas detection technique has extremely broad application in many fields. However, the laser sources currently used in PAS limit the sensing performance. Compared to diode laser and quantum cascade laser, the solid-state laser has the merits of high optical power, excellent beam quality, and wide tuning range. Here we present a long-wave, high-power, wide-tunable, single-longitudinal-mode solid-state laser used as light source in a PAS sensor for trace gas detection. The self-built solid-state laser had an emission wavelength of ~2 μm with Tm:YAP crystal as the gain material, with an excellent wavelength and optical power stability as well as a high beam quality. The wide wavelength tuning range of 9.44 nm covers the absorption spectra of water and ammonia, with a maximum optical power of ~130 mW, allowing dual gas detection with a single laser source. The solid-state laser was used as light source in three different photoacoustic detection techniques: standard PAS with microphone, and external- and intra-cavity quartz-enhanced photoacoustic spectroscopy (QEPAS), proving that solid-state laser is an attractive excitation source in photoacoustic spectroscopy.

## Introduction

Trace gas typically refers mixture with analytes in volume concentrations ranging from ppt (part-per-trillion, 10^−12^) to ppm (part-per-million, 10^−6^). Selective and sensitive trace gas monitoring embraces different applications, spanning from early fire alarm^1,2^ to medical diagnosis^3,4^, together with air pollutant detection, which has caused severe environmental issues, such as the greenhouse effect^5–8^ and photochemical smog^9–11^. Improving the sensitivity of trace gas detection is of great significance for the further developments of these applications.

Up to now, many different spectroscopic methods for trace gas detection have been reported^12–18^ among them, photoacoustic spectroscopy (PAS) is an indirect absorption technique that combines photoacoustic effect with laser absorption spectroscopy. PAS can achieve high detection sensitivity, fast response time, and long-term stability^19–23^. In PAS, an intensity-modulated laser light is tuned at a specific wavelength resonant to an infrared radiative transition of the gas target. As a consequence, the gas molecules absorb photon energy to be excited to a high-energy state. Subsequently, these excited state molecules return to the ground state, with a local release of heating which in turn causes a volumetric expansion. Being the laser light modulated, the volume of the gas will undergo periodic expansion and contraction, resulting in a pressure wave, namely a sound wave. In standard PAS, these weak acoustic waves are detected by a capacitive microphone placed in a resonant photoacoustic cell^24–27^ which should be properly designed in order to have standing wave amplification that make it effective for enhancing photoacoustic signal intensity.

In 2002, a variant of PAS named quartz-enhanced photoacoustic spectroscopy (QEPAS) was reported for the first time^28^. A quartz tuning fork (QTF) was introduced into PAS to replace the microphone as the acoustic wave transducer. When the laser beam is focused between the QTF’s prongs and the frequency of the sound wave matches the fundamental flexural mode of the QTF, the prongs will put into vibrations^29^. Due to piezoelectric nature of the quartz, a polarization field will be generated. The gold electrodes deposited on both prongs collect charges and an electrical signal proportional to the concentration of the absorbing molecules will be generated. Compared with the conventional PAS, millimeter-size QTF easily lends to the miniaturization and integration of the sensor system^30,31^, with the advantages of low cost, narrow response bandwidth as well as a high-quality factor^32,33^. Although in early stages standard commercial QTFs with a resonant frequency of ~32 kHz^34–36^ were widely used in QEPAS, recently custom-made QTFs with low frequency (<10 kHz) demonstrated better performance for photoacoustic detection due to their long energy accumulation time^37–43^.

According to Beer-Lambert’s law, the laser energy absorbed by the target analyte is proportional to the incident laser power. So that a high-power laser is beneficial for improving the photoacoustic signal. At present, light excitation sources commonly used in PAS are distributed feedback (DFB) diode lasers and quantum cascade lasers (QCLs). DFB diode lasers have typical output power of tens of milliwatts^44–46^ and QCLs usually have poor beam quality and harsh working requirements due to the short cavity length and fragile chip^47,48^. Both source types share a narrow wavelength tuning range, limiting a multi-gas detection with a single laser source^49^. In this context, solid-state laser can overcome this limitation thanks to its wide tuning range which enables multi-gas detection with a single laser source. Furthermore, solid-state laser has the advantages of high output power and good beam quality. However, there are absorption lines of multiple gases within the output spectral range of the solid-state laser, so in PAS, to avoid the influence of other gases on the target gas detection, the single longitudinal mode (SLM) output is crucial and can be achieved by putting etalon into laser resonant cavity. In addition, by adjusting the angle of etalons, the wavelength of SLM laser can be tuned to match the gas absorption peak^50^.

In this paper, we demonstrated dual-gas PAS technique employing a long-wave, high-power, wide-tunable, single-mode solid-state laser as gas excitation source. Tm:YAP crystal was used as the laser gain material, and the stable output of a SLM for this solid-state laser was achieved with a double-etalon scheme. The output wavelength of this Tm:YAP laser with SLM was coarse- and fine-tuned by changing the orientation of the double-etalon and the crystal operation temperature, respectively, in order to match the absorption band of water vapor (H_2_O) and ammonia (NH_3_) nearby the 2 μm spectral range. H_2_O detection can ensure the lifespan of sealed electronic devices and prevent corrosion of some special materials, while NH_3_ detection is also significant in medical diagnosis, ensuring production safety and avoiding environmental pollution. The solid-state laser was used as light source in: (i) a conventional PAS setup employing a microphone as acoustic detector; (ii) an external-cavity QEPAS system employing a custom-made QTF in off-beam resonant configuration; and (iii) an intra-cavity QEPAS system, where the QTF was located inside the laser cavity due to its advantage of small size.

## Results

### Solid-state laser characteristics

**Fig. 1:**
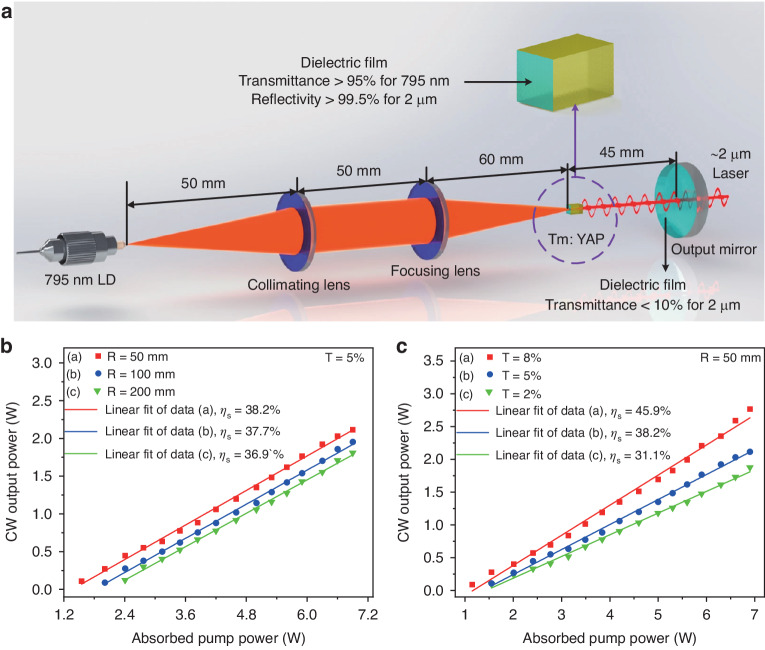
Self-build solid-state laser and optimization of output mirror. **a** Schematic diagram of solid-state laser under free operation mode. LD, laser diode. **b** Optimization of output mirror curvature radius (*R*) with the transmittance (*T*) of 5%. **c** Optimization of output mirror transmittance (*T*) with the curvature radius (*R*) of 50 mm

The self-build solid-state laser in free-running mode is shown in Fig. 1a. A 795 nm fiber-coupled laser diode was used as the pump source. A system of two convex lenses with focal lengths of 50 mm and 60 mm were used to focus the pump laser on Tm:YAP crystal, selected as the gain material, with a geometric dimension of 3 × 3 × 5 mm^3^ and doping concentration of 3.0 at.%. The Tm:YAP crystal absorbed 81% of the incident pump power. The oscillating light transmitted by the crystal is back-forwarded by a plane-concave mirror coated with a dielectric film for 2 μm wavelength, acting as the output mirror of the laser cavity. Moreover, a dielectric film with a transmittance >95% for 795 nm and reflectivity >99.5% for 2 μm was coated on the surface of the crystal forming the front mirror of the laser cavity. Due to the need to achieve a SLM output, and so for a large free spectral range, the cavity length of the solid-state laser should be as short as possible. However, considering the need to insert detection units into the laser resonant cavity in subsequent intra-cavity gas detection experiments, the cavity length cannot be set too short, which finally was set to 45 mm.

The key parameter for evaluating the output characteristics of solid-state lasers is the slope efficiency, defined as the output power per unit of absorbed pump power. Therefore, the slope efficiency has been studied as a function of the curvature radius and transmittance of the output mirror. The continuing wave (CW) output power as a function of absorbed pump power with different curvature radius and transmittance of the output mirror were measured and plotted in Fig. 1b and Fig. 1c, respectively. According to the results, the slope efficiency deteriorates for radii of curvature higher than 50 mm, as for low transmittance of the output mirror. For this reason, the output mirror having a curvature radius of 50 mm and a transmittance of 8% was selected, guaranteeing a slope efficiency of 45.9% to achieve high output power for the subsequent single longitudinal mode laser.

**Fig. 2:**
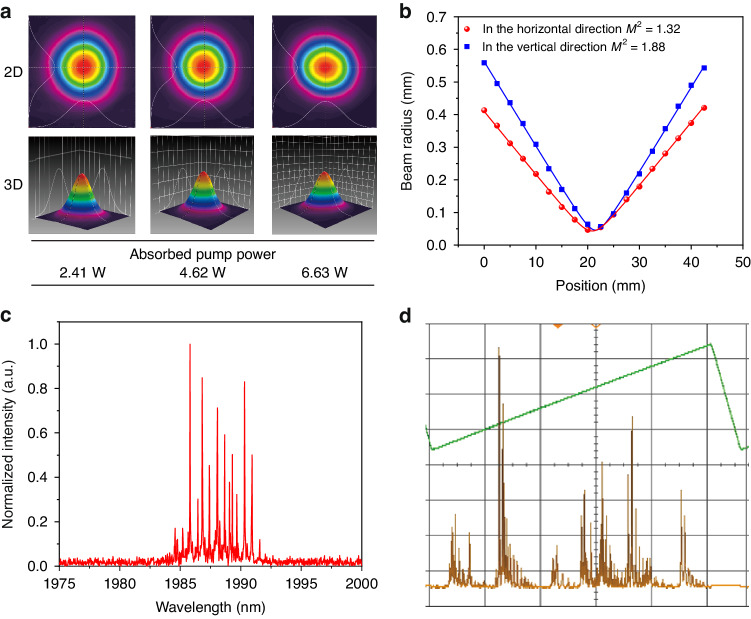
Output characteristics of the solid-state laser under free operation mode. **a** Laser beam profile with different absorbed pump power measured by a CCD camera. **b** Measurement of laser beam quality factor M^2^ for the solid-state laser under free operation exploiting the knife-edge method. **c**, **d** Output spectrum of the solid-state laser under free operation measured with a wavelength meter and a Fabry–Perot interferometer, respectively

The beam profiles of the output laser in free-running operation were acquired at different absorbed pump powers by using a CCD camera. As shown in Fig. 2a, the beam spatial profile resembles an excellent Gaussian distribution even at absorbed pump power as high as 6.63 W. The beam quality was evaluated by measuring the beam quality factor M^2^ with the knife-edge method. The results are depicted in Fig. 2b. The data were fitted with the hyperbolic equation, resulting in M^2^ = 1.32 and 1.88 in the horizontal and vertical direction, respectively. A wavelength meter and a Fabry–Perot interferometer were used to measure the output spectrum of the solid-state laser. The obtained results are shown in Fig. 2c and Fig. 2d, respectively. The spectrum exhibits multiple longitudinal modes in the spectral range from 1982 to 1991 nm under free operation, providing a good starting point for a wide spectral tunability for the subsequent SLM laser.

To be used as light source in a photoacoustic sensor, the SLM output was achieved by using a double-etalon system. When a single etalon is inserted into the solid-state laser cavity, only laser modes that spectrally match the transmission peaks of etalon can be amplified, while all other modes without transmission overlapping are relatively suppressed. However, due to the broad gain spectrum of the selected Tm:YAP crystal and the strong overlap between adjacent longitudinal modes (see Fig. 2c), the free spectral range (FSR) and the finesse of a single etalon cannot meet the requirements for a spectrally pure SLM output. For this reason, a double-etalon system inserted into the laser cavity has been tested. The transmission spectra of the two etalons are superimposed on each other, resulting in an improvement of the spectral filtering at the laser output.

**Fig. 3:**
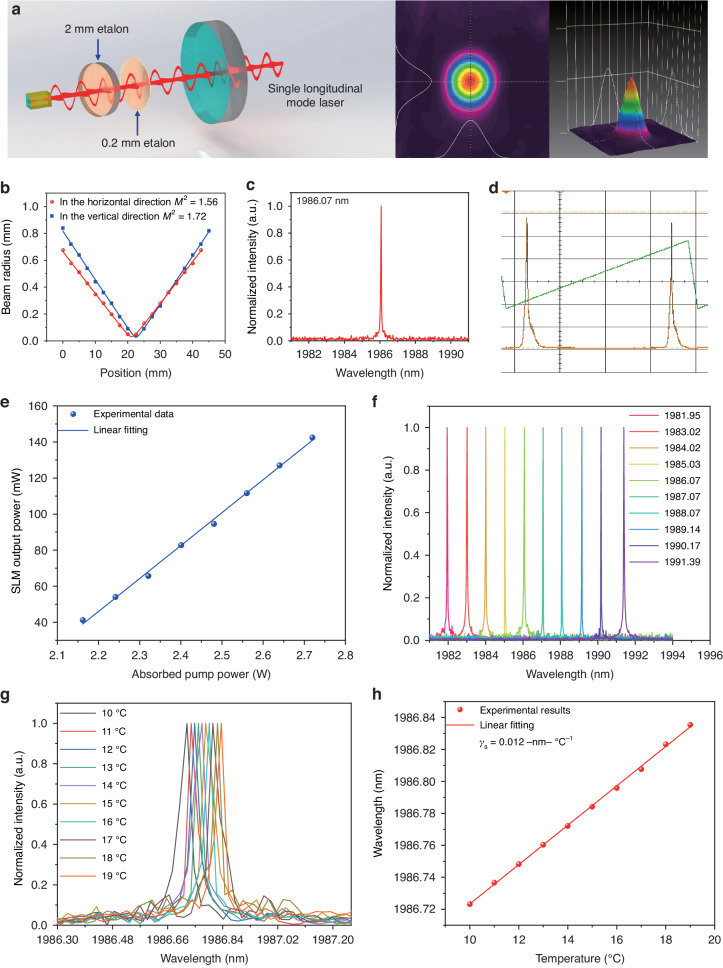
Solid-state laser under single longitudinal mode (SLM). **a** Schematic diagram of solid-state laser under SLM output and the measured single-mode laser beam profile. **b** Measurement of beam quality factor M^2^ for SLM laser exploiting the knife-edge method. **c**, **d** Output spectrum of the solid-state laser under SLM measured with a wavelength meter and a Fabry–Perot interferometer, respectively. **e** The SLM output laser power as a function of pump power fitted with a linear function. **f** The coarse-tuning of the SLM laser wavelength by adjusting the relative angle of two etalons. **g** The fine-tuning of the SLM laser wavelength by adjusting the operation temperature of the Tm:YAP crystal. **h** The variation of SLM laser wavelength following the crystal operation temperature with a linear fitting

The double-etalon scheme is depicted in Fig. 3a. Two etalons with thickness of 0.2 and 2 mm were inserted into the laser resonant cavity. A fine tilting of relative angle between the two etalons allowed the optimization of spectral purity of the single mode output. The beam profile acquired with the CCD camera demonstrates that the double-etalon does not alter the beam quality at the laser output, preserving an excellent Gaussian intensity distribution, as demonstrated by a measured beam quality factor M^2^ of 1.56 in the horizontal direction and 1.72 in the vertical direction (Fig. 3b). The spectrum of the SLM laser was recovered by a wavelength meter and a Fabry–Perot interferometer, as shown in Fig. 3c and Fig. 3d, respectively. With the spectra acquired by the Fabry–Perot interferometer, the linewidth of the laser output mode was estimated to be approximately 1.85 pm (141.13 MHz) which is far less than the full-width half-maximum of a gas absorption line, even at pressures as low as few tens of torr. The output power of SLM laser as a function of absorbed pump power was investigated and the results reported in Fig. 3e. The output power linearly increased with the absorbed pump power: with the absorbed pump power was 2.72 W, the output power of SLM laser reached the maximum of 142.51 mW, which is much higher than typical power levels provided by commercially available diode lasers usually employed in PAS sensors. However, at absorbed pump power levels higher than 2.72 W, other longitudinal modes will reach their threshold simultaneously, deteriorating the spectral purity of the SLM output. Thus, hereafter the absorbed pump power was set to 2.72 W to guarantee a high-power stable output of SLM. By adjusting the relative angle of two etalons, the wavelength of SLM laser can be coarse-tuned over a wide spectral range of 9.44 nm, as shown in Fig. 3f. A fine-tuning of the SLM laser wavelength was achieved by varying the temperature of the Tm:YAP crystal, properly mounted on a thermoelectric cooler (TEC). The spectral emission of the SLM laser at different crystal temperatures is shown in Fig. 3g. For each spectrum, the peak value was extracted and plotted as function of the crystal temperature in Fig. 3h. A linear fit of the experimental data returns a tuning coefficient (γ_s_) of 0.012 nm °C^−1^.

### Solid-state laser-based PAS sensor

**Fig. 4:**
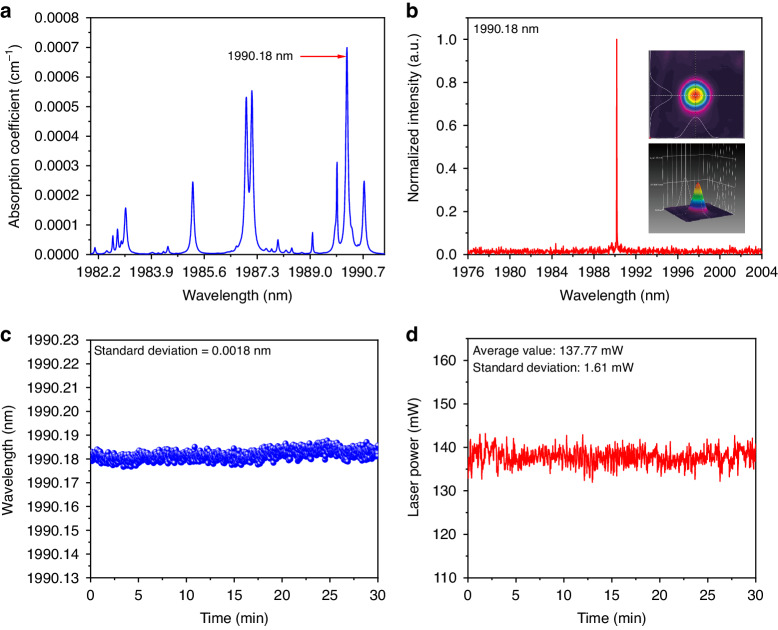
Output characteristics of SLM solid-state laser matched with the selected H_2_O line. **a** Absorption spectra of H_2_O in the SLM laser tunable range. **b** Spectrum and beam profile of the SLM laser for the detection of H_2_O. **c**, **d** Continuous 30-min measurement of the wavelength and output power for the SLM laser matched with selected H_2_O absorption line

According to HITRAN database^51^, the wavelength tunable range of this SLM solid-state laser covers the absorption spectra of H_2_O and NH_3_. In the following experiment, H_2_O was detected first. The absorption coefficient of H_2_O at atmospheric pressure in the SLM laser tunability range is shown in Fig. 4a. The strongest absorption line located at 1990.18 nm was chosen as the target line. By adjusting both the relative angle between the two etalons and the crystal operation temperature, the wavelength of SLM laser was tuned to the gas absorption peak. The spectral emission of the SLM was measured by the wavelength meter and shown in Fig. 4b together with the laser beam profile in the inset. To test the long-term stability of the laser source, the wavelength and output power were continuously monitored for 30 min, as shown in Fig. 4c and Fig. 4d, respectively. With a relative 1σ-standard deviation of 0.9 ppm and 1.1%, the laser source exhibits an excellent stability in wavelength and power output, respectively.

**Fig. 5:**
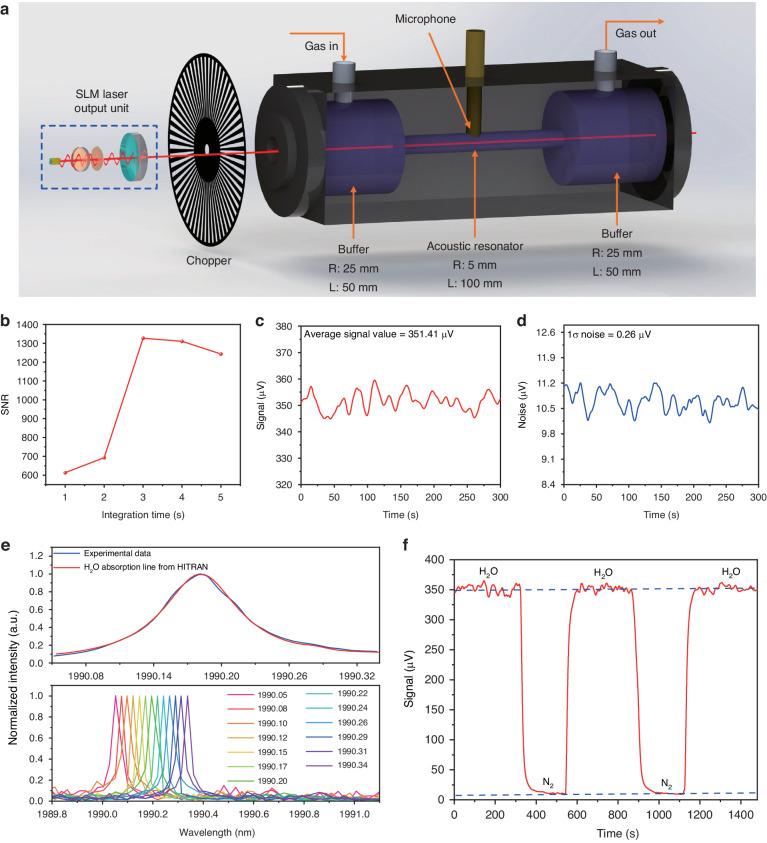
Solid-state lase-based H_2_O-PAS detection. **a** Schematic diagram of the structure for the SLM solid-state laser-based PAS sensor system. **b** Optimization of integration time for the H_2_O-PAS system under the H_2_O concentration of 11,493.05 ppm. **c**, **d** The measured photoacoustic signal and noise of the H_2_O-PAS system under the integration time of 3 s. **e** The comparison between the inverted H_2_O absorption line by the experiment and the standard absorption line from the HITRAN database, and a part of the SLM laser measured by the wavelength meter during the tuning process. **f** The variation of the detected signal by cyclically filling H_2_O and pure N_2_ into the photoacoustic cell

The SLM solid-state laser-based PAS sensor configuration is shown in Fig. 5a. The output laser beam was intensity-modulated by means of a mechanical chopper and injected into a resonant photoacoustic cell consisting of two buffers with a radius of 25 mm and length of 50 mm configured on both sides of a one-dimensional resonant tube photoacoustic tube, to reduce the noise from gas flow. The radius and length of the resonant tube are 5 and 100 mm, respectively. A capacitive microphone was placed at the center of the resonant tube, where the antinode of the one-dimensional resonant mode is supposed to be located. The photoacoustic cell exhibits a resonance frequency of 1579.63 Hz with a bandwidth of 109.71 Hz, corresponding to quality factor of ~14 at atmospheric pressure. Therefore, the chopping frequency was set at the resonance frequency of the cell.

With a mixture containing H_2_O concentration of 11,493 ppm flowing in the photoacoustic cell, the signal-to-noise ratio (SNR) was measured at different integration times *τ*, as shown in Fig. 5b. Starting from *τ* = 1 s, the SNR rapidly increases up to 1351.58 at *τ* = 3 s. For higher integration times, the SNR starts to deteriorate. Thus, *τ* = 3 s was fixed for all other measurements. With the laser wavelength locked to the H_2_O absorption peak, Fig. 5c and Fig. 5d report the PAS signal acquired when the mixture with 11,493 ppm of H_2_O and pure N_2_ flows in the photoacoustic cell, respectively. Divide the gas concentration by the signal-to-noise ratio of the sensor system, the minimum detection limit (MDL) of H_2_O for this solid-state laser-based PAS system was calculated to be 8.7 ppm. The absorption spectral profile of the selected H_2_O line was reconstructed by scanning the SLM laser between 1990.05 nm and 1990.34 nm. The obtained result is shown in Fig. 5e: the experimental data show a good match with the simulated spectral provided by HITRAN database. A part of the SLM laser during the tuning process is also depicted in Fig. 5e. The response time of the PAS sensor as well as the reproducibility of the measurement were evaluated by cyclically filling H_2_O and pure N_2_ into the photoacoustic cell, as shown in Fig. 5f, indicating a good stability of the reported system.

**Fig. 6:**
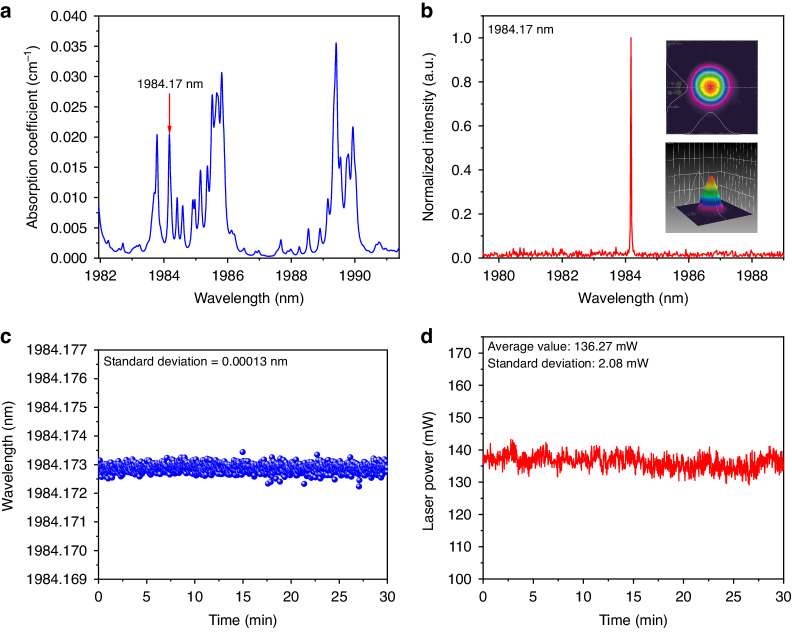
Output characteristics of SLM solid-state laser matched with the selected NH_3_ line. **a** Absorption spectra of NH_3_ in the SLM laser tuning range. **b** Spectrum and beam profile of the SLM laser for the detection of NH_3_. **c**, **d** Continuous 30-minute measurement of the wavelength and output power for the SLM laser matched with selected NH_3_ absorption line

The same sensor architecture depicted in Fig. 5a can be used to target the NH_3_ whose absorption spectrum within the SLM laser dynamic range is shown in Fig. 6a. To test the sensor, the gas absorption line located at 1984.17 nm was selected, free from H_2_O interferents (see Fig. 4a). The same procedure was adopted as for the H_2_O line detection: the SLM laser wavelength was tuned to match the selected line and the beam profile was measured (Fig. 6b), then the laser wavelength and optical power stability were verified with a 30-min acquisition at *τ* = 3 s with the lasers locked at 1984.17 nm, as shown in Fig. 6c and Fig. 6d, respectively.

**Fig. 7:**
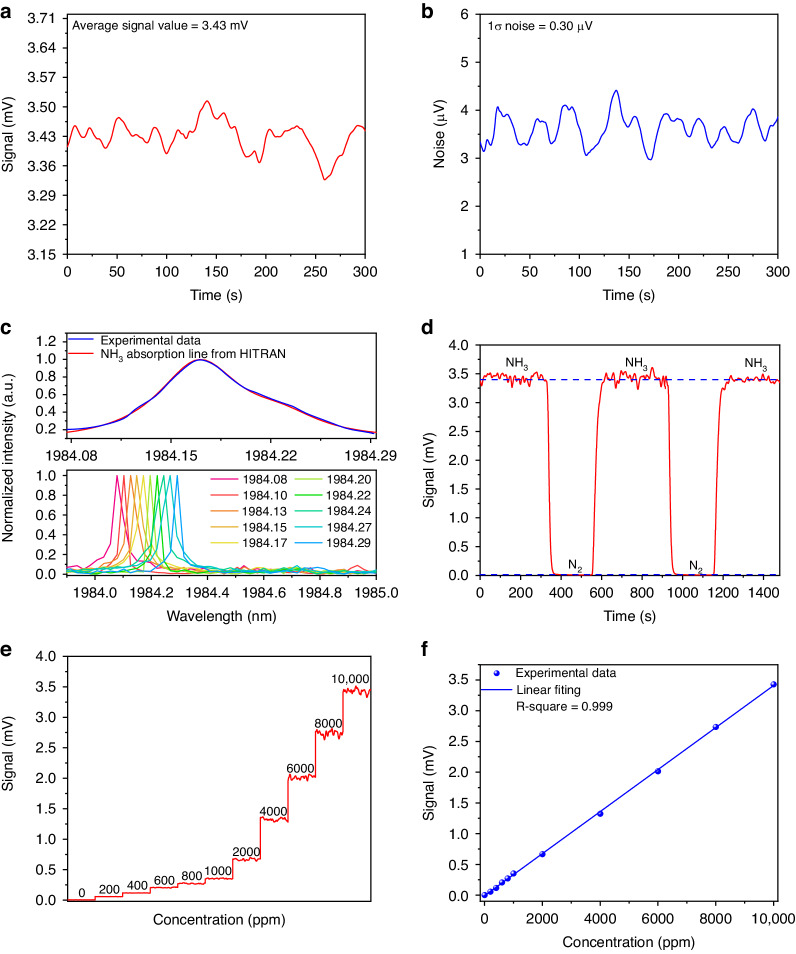
Solid-state laser-based NH_3_-PAS detection. **a**, **b** The measured photoacoustic signal and noise of the NH_3_-PAS system under the integration time of 3 s. **c** The comparison between the inverted NH_3_ absorption line by the experiment and the standard absorption line from the HITRAN database, and a part of the SLM laser measured by the wavelength meter during the tuning process. **d** The variation of the detected signal by cyclically filling NH_3_ and pure N_2_ into the photoacoustic cell. **e** Photoacoustic signal under different NH_3_ concentrations. **f** Photoacoustic signal amplitude as a function of NH_3_ concentrations exploiting a linear fitting

Consequently, the PAS signal was recorded with the cell filled with 10,000 ppm NH_3_:N_2_ mixture (Fig. 7a) and pure N_2_ (Fig. 7b). Based on the measured results, a SNR of 11,433 and a MDL of 875 ppb for the NH_3_-PAS sensor was achieved. Finally, the selected NH_3_ absorption line was reconstructed by fine-tuning the wavelength of the SLM laser. The result is shown in Fig. 7c. Compared with the simulated absorption feature from the HITRAN database, the experimental result perfectly matches it. Figure 7d reports the analysis of repeatability of the sensor, acquired alternating a flow of 10,000 ppm NH_3_:N_2_ mixture (Fig. 7a) with that of pure N_2_. A good signal stability of the NH_3_-PAS sensor system was achieved based on the results shown in Fig. 7d. The sensitivity was evaluated by measuring the PAS signal at different NH_3_ concentrations, obtained by diluting the certified concentration of 10,000 ppm NH_3_:N_2_ with pure N_2_. Figure 7e depicts the step-wise calibration where the PAS signal was acquired for 300 seconds for each NH_3_ concentration with the laser wavelength locked at 1984.17 nm. For each NH_3_ concentration, the mean value was extracted and plotted as a function of NH_3_ concentration in Fig. 7f. The linear fit of the experimental data retrieves a sensitivity of 3.42e^−4^ mV ppm^−1^. A high R-square value of 0.999 was achieved, which means the reported sensor has an excellent linear gas concentration response.

### Solid-state laser-based external-cavity QEPAS sensor

**Fig. 8:**
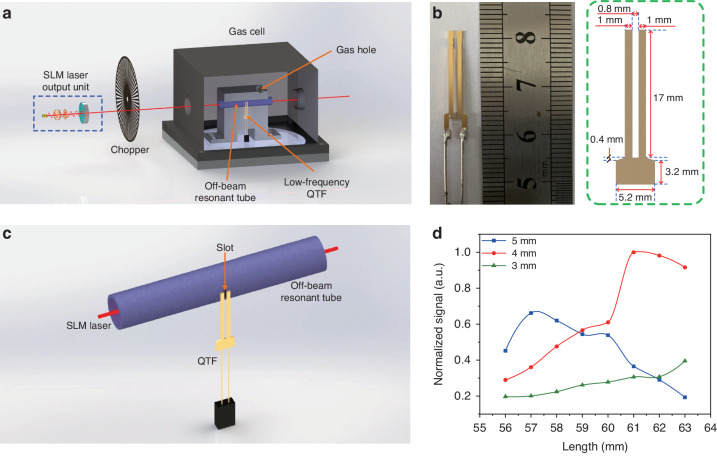
Solid-state laser-based external-cavity QEPAS detection. **a** Schematic diagram of the structure for the single-mode solid-state laser-based external-cavity QEPAS sensor system. **b** Physical image and geometric parameters of the self-designed low-frequency quartz tuning fork (QTF). **c** Schematic diagram of the structure for the off-beam configuration. **d** Optimization of the radius and length for the off-beam resonant tube

Due to the advantages of small geometric dimension, high-quality factor and ability to resonate with sound waves, quartz tuning fork (QTF) was used to construct a QEPAS sensor. The intensity-modulated SLM solid-state laser was coupled with a QEPAS cell for realizing an external-cavity QEPAS sensor, as schematically depicted in Fig. 8a. The QEPAS cell has a volume of 130 mm × 130 mm × 90 mm and contains a spectrophone composed by a custom QTF with a low resonant frequency *f*_0_ = 2890 Hz (geometry and size are reported in Fig. 8b) and a resonator tube in off-beam configuration, sketched in Fig. 8c. In the off-beam configuration, the laser passes through the tube and excites the gas to generate a PAS signal in the tube, which has the advantageous of avoiding thermal noise introduced by laser irradiation on the surface of the QTF. A slit is opened in the middle of the tube where the antinode point of the standing wave pattern is supposed to be located. The width of the slit opened in the middle of the tube was 1 mm with a cut depth of about 1.3 mm. The QTF is aligned to the tube in order that the slit is coincident with the prongs’ gap of the QTF (see Fig. 8c). Due to small diameter, the resonator tube can be considered as a one-dimensional acoustic resonator. To generate a fundamental standing wave vibrational pattern with the antinode point at the center of the resonator tube (where the slit is located), the length of the tube should be an integer multiple of half wavelength of the sound wave. Assuming a speed of sound of *v* = 343 m s^−1^ and being the sound wave generated at the frequency of QTF, the optimal tube length L must be equal to *L* = *v*/2*f*_0_ = 59 mm. Different tube lengths ranging from 56 mm to 63 mm with a step of 1 mm were experimentally tested. For the radius, an excessive radius will prevent the tube from working in a one-dimensional resonant mode, and an excessively small radius can easily lead to laser irradiation on the inner wall of the tube, introducing thermal noise to the system. Therefore, the radius was set as 3 mm, 4 mm, and 5 mm, respectively. The midpoint of the slit on the off-beam tube was set to 1.5 mm from the top of the QTF, which is verified as the optimal position in a previous work^52^. The off-beam resonant tube optimization study was conducted by selecting H_2_O as the target gas. The results are shown in Fig. 8d, where data are normalized to the maximum signal amplitude, corresponding to the resonator tube having a length of 61 mm and a radius of 4 mm.

**Fig. 9:**
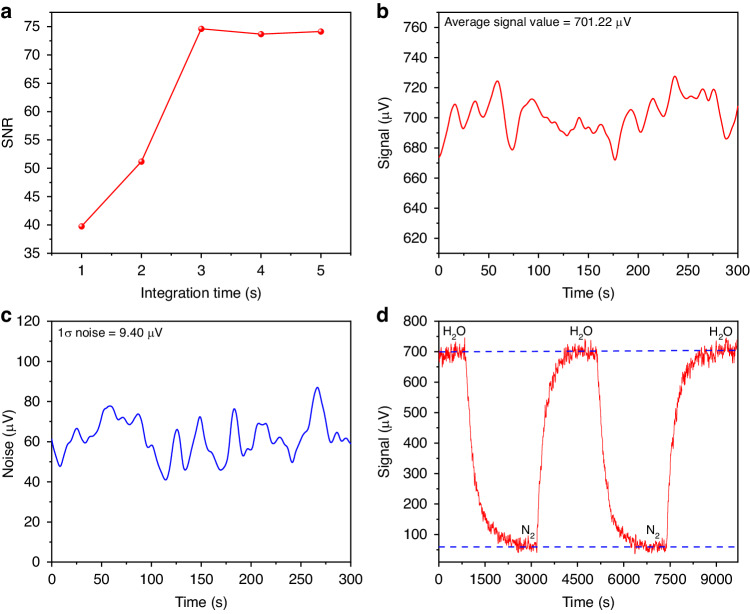
Solid-state laser-based external-cavity H_2_O-QEPAS detection. **a** Optimization of integration time for external-cavity H_2_O-QEPAS system under H_2_O concentration of 4276.02 ppm. **b**, **c** The measured photoacoustic signal and noise of external-cavity H_2_O-QEPAS system under the integration time of 3 s. **d** The variation of the detected signal by cyclically filling H_2_O and pure N_2_ into the gas cell

As did for the PAS system, the external-cavity QEPAS sensor was tested for targeting H_2_O at 1990.18 nm and NH_3_ at 1984.17 nm, in sequence. With a H_2_O concentration of 4276.02 ppm, the optimum integration time for the external-cavity QEPAS system was found to be 3 s, as shown in the trend plotted in Fig. 9a. Figure 9b and Fig. 9c report the external-cavity QEPAS signal when the humidified mixture at 4276.02 ppm and pure N_2_ flows in the QEPAS cell, respectively. After calculation, the MDL of the SLM solid-state laser-based external-cavity QEPAS system is 57.3 ppm for the detection of H_2_O. The performance of the external-cavity QEPAS system is not as good as that of the PAS system. The main reason can be ascribed to the noise introduced by the instabilities of the chopper modulation frequency. Due to the higher resonance frequency of QTF (2890 Hz) compared to the photoacoustic cell in the PAS system (1580 Hz), a faster rotary speed of the chopper introduces stronger external vibrations within the bandwidth of the QTF, compromising the noise level of the external-cavity QEPAS system. Furthermore, the frequency of such vibration matches the resonant frequency of the QTF. So the QTF can respond to this vibration, resulting in a large bias signal in the noise for the external-cavity QEPAS system as shown in Fig. 9c. Because of the extremely narrow response bandwidth of the QTF, the instability of the chopper modulation frequency will cause significant fluctuations in such bias signal which correspond to the noise of the external-cavity QEPAS system. Finally, the QEPAS signal was continuously measured by periodically switching from H_2_O to pure N_2_ into the gas cell (Fig. 9d). The results proved the external-cavity H_2_O-QEPAS sensor achieved good signal stability. After calibration, the gas cell can be removed, and expose the detection unit to the measured environment. In that condition, the signal stabilization time can be significantly improved.

**Fig. 10:**
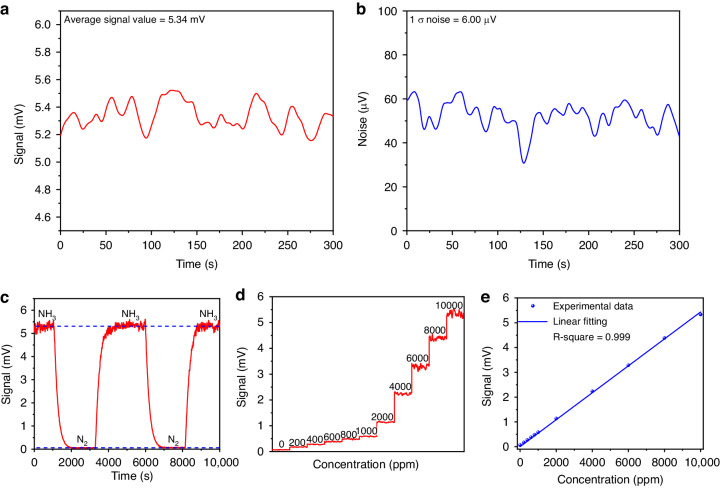
Solid-state laser-based external-cavity NH_3_-QEPAS detection. **a**, **b** The measured photoacoustic signal and noise under the integration time of 3 s. **c** The variation of the detected signal by cyclically filling NH_3_ and pure N_2_ into the photoacoustic cell. **d** Photoacoustic signal measured under different NH_3_ concentrations. **e** Photoacoustic signal amplitude as a function of NH_3_ concentrations exploiting a linear fitting

Switching to NH_3_ detection, the external-cavity QEPAS signals were acquired with 10,000 ppm NH_3_:N_2_ and pure N_2_, and reported in Fig. 10a and Fig. 10b, respectively. The signal measured by circulating 10,000 ppm NH_3_ and pure N_2_ into the gas cell is shown in Fig. 10c, which proved the good signal stability for the external-cavity NH_3_-QEPAS system. Stepwise concentration measurements were performed to verify the linearity of the QEPAS signal as a function of the NH_3_ concentration. The QEPAS signal for each concentration step was measured every 300 s and the results are shown in Fig. 10d. Data for each step were averaged and a calibration curve was obtained in Fig. 10e as the best linear fit of the experimental data. It can be seen the reported SLM solid-state laser-based external-cavity NH_3_-QEPAS achieved an excellent linear concentration response. Finally, a MDL of 11.2 ppm was estimated, which is worse than the performance recorded with the PAS sensing system and the reason should be same as the external-cavity H_2_O-QEPAS.

### Solid-state laser-based intra-cavity QEPAS sensor

Being the sensitivity of the PAS technique linearly dependent by the optical power, including the PAS cell within the cavity would be exploited the extremely high intra-cavity optical power density. However, in order to achieve SLM laser oscillation in the cavity, the length of the cavity should be short to increase the longitudinal mode interval of the laser. This leads to difficulty in placing most acoustic detectors with bulky sizes in the laser cavity. However, the QTF has millimeter-size (Fig. 8b), which makes it possible to achieve intra-cavity gas detection.

**Fig. 11:**
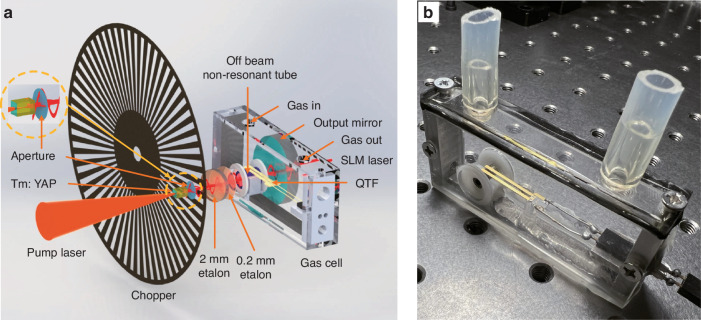
Solid-state laser-based intra-cavity QEPAS gas sensor. **a** Schematic diagram of the structure for the solid-state laser-based intra-cavity QEPAS sensor system. **b** Physical image of the designed gas cell with a non-resonant off-beam configuration

The schematic of the SLM solid-state laser-based intra-cavity QEPAS sensor is shown in Fig. 11a. An aperture was put inside the laser resonant cavity first and close to the Tm:YAP crystal to avoid the influence of the pump laser. To intensity-modulate the intra-cavity optical power, the chopper is placed between the aperture and the 2-mm-thick etalon, far from the QTF in order to reduce the extra-acoustic noise generated by the high rotary speed of the chopper. A picture of the QEPAS cell with a volume of 70 mm × 14 mm × 31 mm is shown in Fig. 11b. The QEPAS spectrophone is arranged in off-beam configuration as before, with the tube length set as 10 mm due to the space limitations within the optical cavity. In such condition, the tube can only work in a non-resonant mode.

**Fig. 12:**
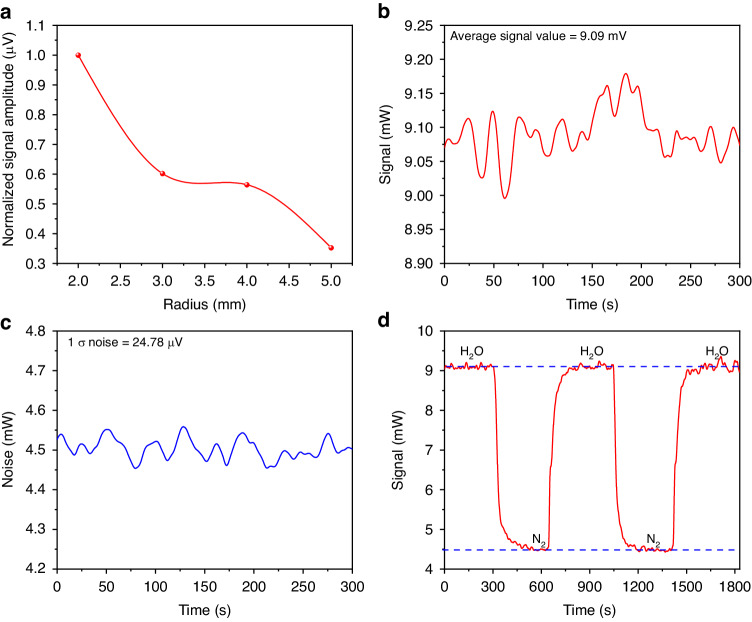
Solid-state laser-based intra-cavity H_2_O-QEPAS detection. **a** Optimization for the radius of the non-resonant off-beam tube. **b**, **c** The measured photoacoustic signal and noise of the intra-cavity H_2_O-QEPAS system under the integration time of 3 s. **d** The variation of the detected signal by cyclically filling NH_3_ and pure N_2_ into the photoacoustic cell

Different tube radii were tested and results are shown in Fig. 12a. The data are normalized to the higher value, namely the SNR recorded with a radius of 2 mm. It can be seen that as the radius of the tube decreases, the signal amplitude increases. In the experiment, the minimum radius of the tube was 2 mm which is the optimum value. The reason why the radius of the tube was not set smaller is to prevent from irradiation on the inner wall of tube. The intra-cavity QEPAS sensor was tested for targeting H_2_O at 1990.18 nm and NH_3_ at 1984.17 nm, in sequence. First with a H_2_O concentration of 15,053 ppm and then with pure N_2_, the measured QEPAS signal are displayed in Fig. 12b and Fig. 12c, respectively. After calculation a MDL of 41.0 ppm was obtained. The 1σ-standard deviation of the intra-cavity QEPAS signal (24.78 µV) in pure N_2_ is significantly higher than that recorded for external-cavity QEPAS (9.40 µV). On one hand, this is mainly caused by a closer distance between the QTF and the chopper, with respect to external-cavity configuration. The environmental vibration introduced by the chopper has a more serious impact on the QTF. On the other hand, due to the big divergence of the pump light, a portion of the laser was irradiated on the surface of the QTF. The QTF can absorb the laser energy resulting in a thermal expansion. Under the effect of intensity modulation, the QTF generates periodic expansion and contraction eventually producing a piezoelectric signal. Such a large bias signal is also the main reason for the higher noise of the intra-cavity QEPAS system. By circulating H_2_O and pure N_2_ into the gas cell, the signal was continuously measured as shown in Fig. 12d. The obtained results prove that the intra-cavity QEPAS system has good signal stability.

**Fig. 13:**
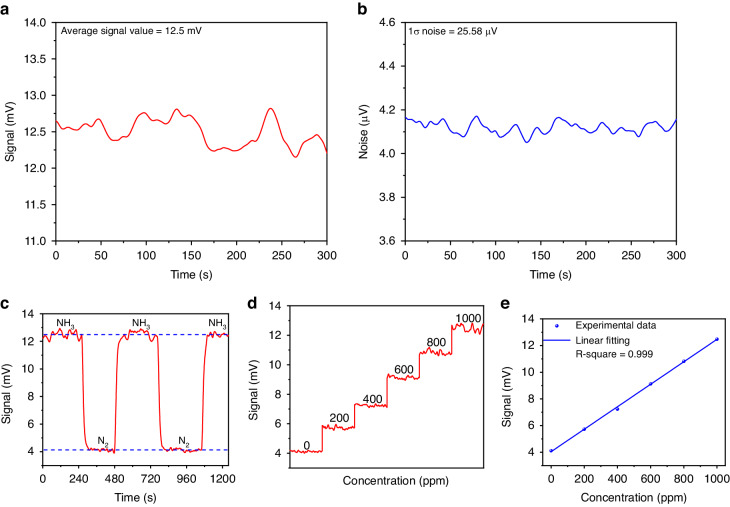
Solid-state laser-based intra-cavity NH_3_-QEPAS detection. **a**, **b** The measured photoacoustic signal and noise under the integration time of 3 s. **c** The variation of the detected signal by cyclically filling NH_3_ and pure N_2_ into the photoacoustic cell. **d** Photoacoustic signal measured under different NH_3_ concentrations. **e** Photoacoustic signal amplitude as a function of NH_3_ concentrations exploiting a linear fitting

To prevent that high NH_3_ concentration cause a huge increase in the intra-cavity loss that in turn can compromise the spectral properties of the cavity (such as the finesse), a mixture with 1000 ppm of NH_3_ was used instead of 10,000 ppm. The measured intra-cavity NH_3_ QEPAS signal is shown in Fig. 13a while that acquired in pure N_2_ is displayed in Fig. 13b. Good signal stability is also proved by measuring the signal with the circulation of NH_3_ and pure N_2_ into the gas cell, which is depicted in Fig. 13c. The sensor was then calibrated with successive dilutions of the 1000 ppm NH_3_ in N_2_ (Fig. 13d) and the sensor response is reported in Fig. 13e, with the best linear fit of the experimental data leading to a sensitivity of 0.00842 mV ppm^−1^. The MDL of the intra-cavity NH_3_-QEPAS system was found to be 2.0 ppm, which is 5.5 times improvement compared to the external-cavity QEPAS sensing system (MDL of 11.2 ppm). For the reported three different systems, all the relationship between the obtained signal and gas concentration shows that the signal fluctuation increases with the increasing of gas concentration. This is mainly because as the concentration increases, the average value of the signal also increases. When the change rate is the same, the signal will exhibit greater fluctuation under high concentration.

## Discussion

This study demonstrated ultra-highly sensitive dual gases detection based on photoacoustic spectroscopy by exploiting a long-wave, high-power, wide-tuning, single-longitudinal-mode solid-state laser. Compared to the laser sources of DFB diode laser and QCL, the solid-state laser self-built in the reported system achieved high optical power (~136 mW) and long output wavelength (~2 μm) while maintaining excellent beam quality and avoiding harsh working conditions. It is much suitable as the excitation source in the PAS which can excite a strong and stable photoacoustic signal thereby achieving good performance for the detection of trace gas. Moreover, the single-longitudinal-mode solid-state laser provided a wide tuning range of 9.44 nm which allows to target absorption spectral features of H_2_O and NH_3_. It demonstrated the capability to perform multi-gas detection using this kind of laser source. Three different photoacoustic spectroscopy-based systems were designed and gas detection performances in the few ppm range were achieved. For the microphone-based PAS sensor, the MDL for H_2_O and NH_3_ detections were determined to be 8.7 ppm and 875 ppb, respectively. For the external-cavity QEPAS sensor, 57.3 ppm and 11.2 ppm were obtained for H_2_O and NH_3_ measurements, respectively. For the intra-cavity QEPAS sensor, the high-power density in the resonant cavity of the solid-state laser was utilized to improve the detection performance. The MDL of 41.0 ppm and 2.0 ppm were achieved for H_2_O and NH_3_ intra-cavity measurements. In the experiment, the performance of the intra-cavity QEPAS system is not as good as that of the PAS system, this is mainly caused by the excessive noise from the rotation of the chopper and irradiation of the pump laser. Such noise can be reduced by changing the laser intensity modulation method or increasing the gap interval of the chopper plates to decrease the rotation speed of the chopper. This study proves that solid-state laser is an attractive excitation source in PAS and the solid-state laser-based intra-cavity QEPAS provides new ideas for the design of PAS systems.

## Materials and methods

### Build of single-longitudinal-mode solid-state laser

A fiber-coupled diode laser with emission wavelength of 795 nm was served as the pumping source. The core diameter of the fiber pigtail is 200 μm and the numerical aperture is 0.22. A Tm:YAP crystal cut in the a-direction with the maximum emission bandwidth was chosen as the gain material for the solid-state laser to obtain the ~2 μm laser. To achieve the SLM output, two etalons with a diameter of 10 mm and thicknesses of 0.2 mm and 2 mm were inserted into the laser resonant cavity. The etalons were not coated. By adjusting the angle of two etalons and changing the crystal operation temperature with a self-designed thermoelectric cooler (TEC), the wavelength of SLM can achieve coarse-tuning and fine-tuning, respectively.

### Laser characteristic measurement setup

A CCD camera (model No. Pyrocam III HR, Spiricon) was utilized to measure the laser beam profile. The knife-edge method was exploited to measure and calculate the beam quality factor M^2^ for the laser during free operation and SLM. The output spectrum of the laser was measured by employing a wavelength meter (model No. 771B-MIR, Bristol) and a Fabry–Perot interferometer (model No. SA210-12B, free spectral region: 10 GHz, Thorlabs), simultaneously.

### Build of the trace gas sensor system

For the microphone-based PAS system, a resonant one-dimensional photoacoustic cell with a radius of 5 mm and a length of 100 mm was used to amplify the photoacoustic signal. A condenser microphone with detection sensitivity of 50 mV Pa^−1^ was installed inside the cell. The resonant frequency of the photoacoustic cell was determined as 1580 Hz. Two buffers with radius of 25 mm and length of 50 mm were set at both sides of the resonant tube aiming to reduce the noise from gas flow.

In the external-cavity QEPAS system, an off-beam resonant configuration was employed. The off-beam tube is made of stainless steel material and the optimum length and radius were 61 mm and 4 mm, respectively. A custom QTF with a resonant frequency of 2890 Hz was exploited as the acoustic transducer. The geometric parameters of the QTF are displayed in Fig. 8b. The midpoint of the slot on the off-beam tube was set to 1.5 mm from the top of the QTF.

For the intra-cavity QEPAS system, due to the space limitations within the laser resonant cavity, the length of the off-beam tube was set as 10 mm. The optimum radius was 2 mm. Similar to the external-cavity QEPAS system, the slot of the off-beam tube was also set to 1.5 mm from the top of the QTF.

The laser optical intensity modulation method was utilized in all three systems which was achieved by a chopper (model No. MC2000B, chopper blade: MC1F60, Thorlabs). Based on the resonant frequency of the acoustic detection unit, the modulation frequency was set at 1580 Hz for the microphone-based PAS system and 2890 Hz for the QEPAS system, respectively. Moreover, the photoacoustic signals detected by the acoustic transducers in each system are eventually demodulated by employing a lock-in amplifier (model No. MFLI DC-500 kHz, Zurich Instruments).
